# The Limitation of Limitations

**DOI:** 10.1016/j.shj.2024.100283

**Published:** 2024-03-01

**Authors:** Anthony DeMaria

**Affiliations:** Judy and Jack White Chair in Cardiology, Sulpizio Cardiovascular Center, University of California, San Diego, La Jolla, California, USA

I have been the editor of one or another medical journal for nearly 20 years. Although I have not counted the exact number of manuscripts overseen during that time, it is almost certainly over 35,000. During that time, I have not encountered a single manuscript that was judged to be perfect as submitted and did not need or benefit from some revision. In my experience, the perfect manuscript does not exist.

Clearly there are at least 2 important variables in assessing the perfection of a manuscript. One is the paper itself, and the second (and equally important in my experience) are the reviewers. Independent of personal conflict, the reviewers are, of course, motivated to show that they have scrutinized the paper and detected even the slightest flaw. Sometimes the issues raised are very minor, seeking further embellishment, or just stylistic. However, usually the critiques raise very relevant issues, attention to which, in a revision, nearly always leads to an improved article. Regardless of the importance of the questions raised in the evaluation, the authors almost invariably thank the reviewers profusely for their insightful comments and do whatever they can to provide an adequate answer. It is uncommon to see an author resort to major rebuttal of a critique. For full disclosure, I follow the same behavior with my own papers.

Which brings me to the topic of this page, which is the matter of limitations for a study. The overwhelming majority of manuscripts have a Limitations section that details the shortcomings of the article of which the authors are aware. Whether this is a proactive action to soften any criticism (better to confess than to be discovered) or a genuine desire to set the paper in context, cannot be determined. However, the limitations delineated are frequently followed by some explanatory information as to why they are not really important.

During the course of their evaluation, the reviewers not only assess the limitations reported but often identify issues that they believe the authors have not addressed. The gravity of all these issues varies considerably and often requires the judgment of a true expert. Not uncommonly, 2 reviewers will identify the same issue, and one will feel it warrants rejection while the other will not assign it such importance. Assuming the manuscript is being considered for publication, it will typically be returned to the authors to address the concerns in a revision.

When the revised manuscript is resubmitted, it is not uncommon that the authors cannot resolve all the questions raised. In such cases, the authors frequently respond by claiming the unresolved issue as a limitation of the study. The implication is that “sure, it’s a problem, but not sufficient to cast doubt on the findings reported.” So the editors are confronted by a manuscript with limitations identified by the authors and reviewers and uncertainty as to how grave a weakness they actually represent. The paper goes back to the original reviewers, but I often worry that the assignment of a problem to the list of limitations often gets lost in the much longer responses to the questions that can be resolved. In my experience, only occasionally does an external consultant say that the limitation is very grave and that adding it to the list of limitations is inadequate to justify publication.

If the editors are often uncertain about the importance of some limitations, this problem is greatly magnified for the general reader. A reader without expertise in the subject matter of a paper is rarely able to place the findings in the proper context of the limitations. For many who have read only the introduction and conclusion, or those who have only read the abstract, the impact of the limitations is completely lost. The danger, of course, is that they come away ascribing more importance to the findings than are warranted, if indeed any importance at all is justified.

Having given this problem some thought over the years, I am still not sure of the remedy. Perhaps the external review consultants should be asked directly if it is adequate to place some unresolved issue in the Limitations section. Or perhaps limitations should be graded as mild, moderate, and severe and designated as such in the manuscript. It would seem to be a good idea to mandate that limitations be included in the abstract and conclusion paragraphs of the manuscript. At least then, the casual reader who is browsing the paper will be informed of the shortcomings. Although I am unsure of the solution, I am concerned that merely designating a problem as a limitation tends to imply that it is not very important. I see this as the major limitation of limitations.

## Funding

The author has no funding to report.

## Disclosure Statement

The author reports no conflict of interest.

